# Application of protein lysate microarrays to molecular marker verification and quantification

**DOI:** 10.1186/1477-5956-3-9

**Published:** 2005-11-10

**Authors:** Anitha Ramaswamy, E Lin, Iou Chen, Rahul Mitra, Joel Morrisett, Kevin Coombes, Zhenlin Ju, Mini Kapoor

**Affiliations:** 1 Department of Molecular Genetics, The University of Texas MD Anderson Cancer Center, Houston, TX 77030, USA; 2 Department of Biostatistics, The University of Texas MD Anderson Cancer Center, Houston, TX 77030, USA; 3 Department of Medicine, Section of Atherosclerosis, Baylor College of Medicine, Houston, TX 77030, USA; 4 Genomics USA Inc., Houston, TX 77054, USA

## Abstract

This study presents the development and application of protein lysate microarray (LMA) technology for verification of presence and quantification of human tissue samples for protein biomarkers. Sub-picogram range sensitivity has been achieved on LMA using a non-enzymatic protein detection methodology. Results from a set of quality control experiments are presented and demonstrate the high sensitivity and reproducibility of the LMA methodology. The optimized LMA methodology has been applied for verification of the presence and quantification of disease markers for atherosclerosis. LMA were used to measure lipoprotein [a] and apolipoprotein B100 in 52 carotid endarterectomy samples. The data generated by LMA were validated by ELISA using the same protein lysates. The correlations of protein amounts estimated by LMA and ELISA were highly significant, with r^2 ^≥ 0.98 (p ≤ 0.001) for lipoprotein [a] and with r^2 ^≥ 0.94 (p ≤ 0.001) for apolipoprotein B100. This is the first report to compare data generated using proteins microarrays with ELISA, a standard technology for the verification of the presence of protein biomarkers. The sensitivity, reproducibility, and high-throughput quality of LMA technology make it a potentially powerful technology for profiling disease specific protein markers in clinical samples.

## Background

Current methodologies used for protein biomarker studies include i) 2-dimensional polyacrylamide gel electrophoresis [2D-PAGE; 1–4]; ii) mass spectrometry [MS; 5–6]; and iii) immunological assays such as enzyme-linked immunosorbent assay and radio-immuno assay [ELISA and RIA; 7–9]. While they are widely used for discovery of protein biomarkers, MS and 2D-PAGE methodologies have been used for the verification of the presence of pre-determined protein biomarkers in few studies. Immunological assays such as ELISA and RIA are most commonly used for the verification of the presence of pre-determined protein biomarkers [7-9]. However, ELISA and RIA are not readily applicable for detection of known disease markers from very small amounts of starting materials, like lysates from cells obtained from clinical samples. A typical test requires up to micrograms of starting samples that are difficult to obtain in most cases. Further, these techniques are not easily adaptable to high throughput applications required for handling a large number of samples simultaneously.

Microarray-based assays [10-12] provide a solution to these problems. A microarray is a collection of spatially addressable probes immobilized on a surface as spots. The increase in throughput is due to the small spot-size on the microarray (~150–200 μdiameter) that allows for a large number of spots per microarray. Thousands of probes (printed on the microarray surface) can be interrogated for a specific target (in solution) in a single microarray experiment. In addition to the high-throughput achieved, microarray assays are highly sensitive and require extremely small amounts of samples. The increase in sensitivity in microarray-based methods is due to the miniature format, which leads to an increase in the signal density [signal intensity/area; 13]. Compared to the micro titer plate format employed in ELISA, a typical microarray spot is more than 25 times smaller. This concentrates the signal density and enhances the signal intensity. The amount of sample required to saturate a microarray spot also decreases in proportion to its surface area and hence typically a few nanograms are sufficient for several microarray experiments. Thus, sensitivity comparable to or exceeding ELISA can be achieved on microarrays using only a fraction (down to 1/1000^th^) of the sample size required for ELISA. The advantage gained by miniaturization, high sensitivity, and high throughput makes protein microarrays a potentially powerful technology for discovery of new markers and detection of known protein markers [14-20].

In the present study we show the development and application of protein lysate microarrays (LMA), also known as reverse phase arrays [21,22] for interrogating multiple human samples for disease-specific protein markers in a single experiment. In LMA, the samples (up to thousands) are immobilized on the surface of the microarray and a single antibody for the protein biomarker is used to screen the samples for its presence. Each antibody on LMA is used in an independent experiment that is focused on profiling expression of a specific protein across all samples printed on the array leading to uniformity of results. Use of a single antibody also allows for the determination of differences in protein marker levels in different patient samples with greater accuracy.

Variations of LMA technology have been used in different applications in recent years [23,24]. Using lysates prepared from laser capture microdissected human samples, reverse-phase arrays methodology have been successfully used to study regulation of pro-survival pathways at the transition from normal prostate epithelium to intraepithelial neoplasia and into invasive prostate cancer [21]. Madoz-Gurpide and coworkers [25] performed liquid phase protein separation of extracts prepared from lung adenocarcinoma A549 cell line and the various fractions were microarrayed and probed with antibodies against specific proteins. Nishizuka and coworkers [22] have recently used reverse-phase arrays to screen compounds for anticancer activity in a panel containing 60 human cancer cell lines. LMA have also been used for multiplexed analysis of proteins where 2 different antibodies were used for binding on the same arrays for verification of the presence of 2 different proteins or to determine the phosphorylation status of the proteins of interest [26,27].

The signal detection method used in most of the above studies was an enzymatic tyramide-based reaction that catalyzes the deposition of multiple dye molecules at the antibody-binding site. A drawback of this method is the inability to control the enzymatic reaction of the deposition of dyes that often results in saturation of the signal intensities of the microarray spots. In a previous report [28], we presented the various issues associated with using a tyramide-based amplification method for signal detection on microarrays. A biotin-streptavidin based (non-enzymatic) signal amplification and detection methodology used in the LMA experiments presented here overcomes the signal saturation problems faced with the enzymatic tyramide-based methodology.

In this report we present the development and validation of LMA for verification of the presence and quantification of predetermined protein markers using antibodies tested for specificity by Western blot analysis. We have performed quality control experiments to evaluate and optimize the LMA methodology. We have applied the optimized LMA methodology for verification of the presence and quantification of protein markers from atherosclerotic samples. The results were highly correlative with those obtained by ELISA using the same lysates (with r^2 ^≥ 0.98 (p ≤ <0.001) for lipoprotein [a] and r^2 ^≥ 0.94 (p ≤ 0.001) for apolipoprotein B100), but required much less amount of samples. This is the first study to show a parallel comparison of protein microarray data with a standard tool like ELISA, for detection of known protein markers.

## Results

We present here the results from experiments performed to test and validate the LMA technology for verification of the presence and quantification of specific-protein biomarker. We chose to use a cell line for the initial quality control experiments for LMA to facilitate economical use of the available patient samples. F9, a murine terato-carcinoma cell line that can be easily cultured was used. This cell line over expresses tumor suppressor protein p53 [29] and therefore we determined the expression level of p53 in protein lysates prepared from F9 cells in these LMA experiments. Lysates prepared from this cell line were used to optimize printing and binding steps on LMA. Normal human plasma samples were also used to show the reproducibility of the data generated. All together, the results from the quality control experiments demonstrate reproducibility of LMA printing, linearity of the different binding steps and signal detection and sensitivity of the LMA methodology. Next, we applied the LMA technology for verification of the presence of and quantification of specific-protein markers from atherosclerotic tissue samples and compared the data generated with those from ELISA experiments.

### Reproducibility in LMA printing

**Figure 1:**
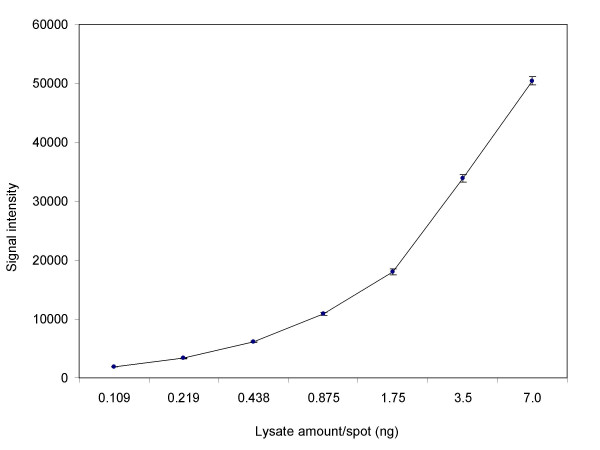
Correlation of signal intensity to lysate amount printed on LMA. Cy3-labeled F9 lysate amount (0.109 – 7.0 ng) printed on LMA was plotted against signal intensity.

One of the major problems in microarray printing is the spot-to-spot variation introduced due to differences in amount of material dispensed. To demonstrate the uniformity of our dispensing (printing) method, we prepared Cy3-labeled protein lysates from F9 cells, made serial dilutions (7 different concentrations) and printed them on slides. The data set consisted of six arrays with 3 replicates per lysate amount per array, resulting in a total of 18 data points per lysate amount. The plot in figure 1 shows a correlative increase in the signal intensity with increase in the printed lysate amount. Table 1 shows a summary of the statistics (mean, standard deviation (SD) and coefficient of variance (CV)) of the data generated. We observed a CV of 2.59–5.47% for the 18 data points per lysate amount printed, indicating high reproducibility in LMA printing. Similar data were generated using Cy5-labeled protein lysates (not shown).

**Table 1:** Summary of data statistics for LMA containing labeled lysates

	**0.109**	**0.219**	**0.438**	**0.875**	**1.75**	**3.5**	**7.0 (ng)**
MEAN	1891.25	3313.75	6152.20	10898.40	18032.50	33889.75	50389.00
SD	76.34	85.92	180.90	444.80	986.24	1197.71	1352.43
CV (%)	4.04	2.59	2.94	4.08	5.47	3.53	2.68

### Linearity of the different binding steps on LMA

**Figure 2:**
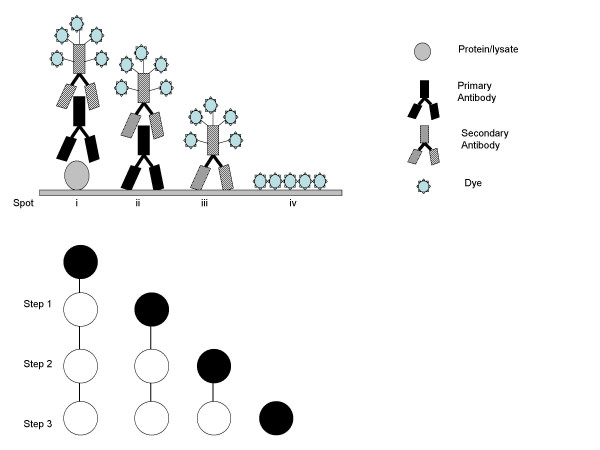
Schematic of the different binding steps on LMA. F9 lysates or purified p53 protein, purified primary p53 antibody, biotinylated secondary antibody, and Cy3-labeled streptavidin were printed on spots i, ii, iii and iv on the LMA, respectively. A diagrammatic representation of the different binding steps performed for signal detection on LMA is shown.

We used a sandwich assay methodology for signal detection on LMA. Lysates prepared from samples were printed on nitrocellulose membrane coated slides and incubated with a specific antibody against the antigen of interest. The arrays were incubated with a biotinylated secondary antibody followed by signal detection using streptavidin linked to Cy3 (or Cy5) dye. The final signal intensity of the LMA spots results from a sequential combination of 3 binding events: 1) binding of the primary antibody to the protein marker in the lysate; 2) binding of the secondary antibody to the primary antibody; and 3) the binding of Cy3-labeled streptavidin to the secondary antibody (Figure 2). To obtain uniform, reproducible and accurate results it is essential to ascertain that each of these binding reactions occurs in a linear fashion.

The linearity of each of the binding reactions on the LMA was determined as follows: LMA with spots containing F9 lysates or purified p53 protein (Figure 2, spot i), purified primary p53 antibody (Figure 2, spot ii), biotinylated secondary antibody (Figure 2, spot iii) and Cy3-labeled streptavidin (Figure 2, spot iv) were printed. In step 1 of binding, LMA was incubated with the primary p53 antibody, in step 2 with biotinylated secondary antibody and finally with Cy3-labeled streptavidin in step 3. In step 1 of binding, the primary antibody will bind to the F9 lysate (that are positive for p53; [29]) and purified p53 protein (Figure 2, spot i). In step 2, the biotinylated secondary antibody will bind to the primary antibody now bound to the F9 lysate and purified p53 protein (Figure 2, spot i), and to purified primary antibody printed on the surface at spot 'ii'. In step 3 the Cy3-labeled streptavidin will bind to secondary antibody generating fluorescent signals at spots 'i', 'ii' and 'iii'. No reactions were expected on spot 'iv' where Cy3-labeled streptavidin alone was printed.

**Figure 3:**
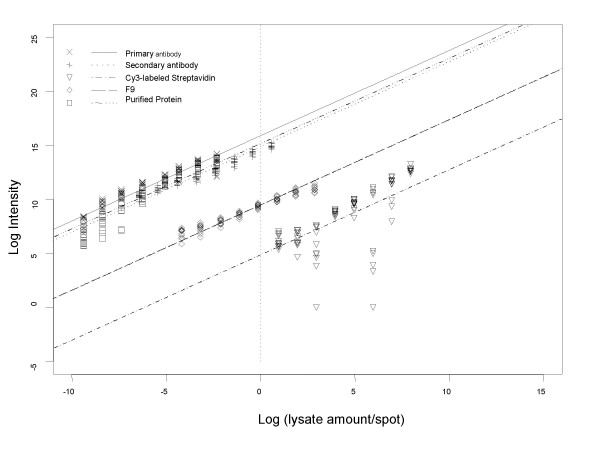
Linearity of different binding steps on LMA. LMA containing serially diluted F9 protein lysates (0.055, 0.11, 0.23, 0.46, 0.92, 1.84, 3.68 and 7.36 ng), purified p53 protein (1.5, 3, 6, 12, 24, 48, 96 and 192 pg), purified primary antibody (1.5, 3, 6, 12, 24, 48, 96 and 192 pg), biotinylated secondary antibody (11.5, 23, 46, 92, 184, 368, 736 and 1472 pg) and Cy3-labeled streptavidin (1.95, 3.9, 7.8, 15.6, 31.2, 62.4, 124.8 and 249.6 ng) were printed. Arrays were probed with p53 antibody followed by binding and labeling steps as described in the Methods. Log transformed (base 2) spotted lysate amount was plotted against log transformed (base 2) signal intensity.

**Figure 4:**
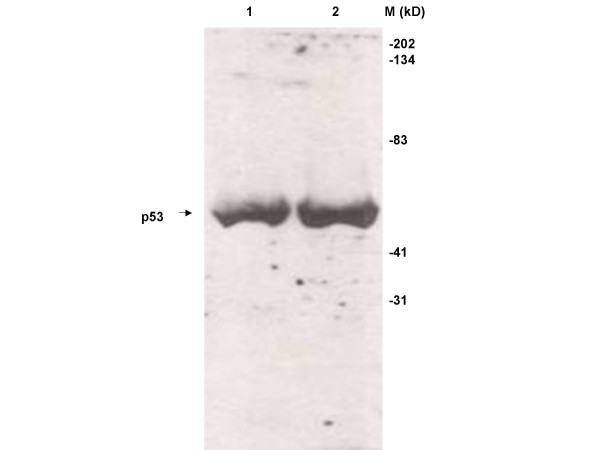
Specificity of p53 antibody. Ten micrograms of extracts from bacteria over-expressing p53 protein (lane 1) and F9 cell lysates (30 μg; lane 2) were analyzed for specificity of the p53 antibody and expression of p53 by Western blot analysis.

Each reagent on the array was printed in a serial dilution. Each dilution of the different reagents was printed in duplicate on the arrays. Two arrays were processed resulting in 4 data-points per concentration per reagent. This experimental design allows determination of the linearity of each binding reaction contributing to the final signal, reproducibility of each reaction and saturation if any, of the binding reactions. The plot in figure 3 shows that all the binding steps described above occurred in a linear and reproducible fashion and did not saturate. Figure 4 shows a Western Blot where extracts from F9 cells (lane 2) were probed for detection of p53 by the same antibody that was used in the LMA experiments. Lysates from bacteria over-expressing p53 protein were used as a positive control for p53 (lane 1). These data demonstrate the expression of p53 protein in F9 cells and the specificity of this antibody as indicated by the unique p53 band observed on the Western blot.

### Reproducibility of signal detection on LMA

**Figure 5:**
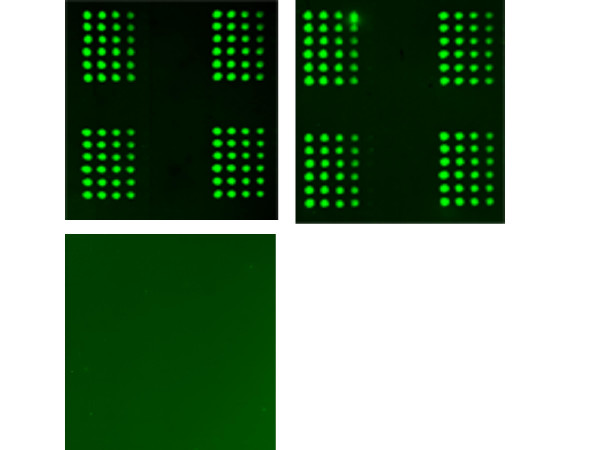
Reproducibility in signal detection on LMA. Serially diluted lysates (5.0 to 0.625 ng) prepared from human plasma sample were printed and probed for detection of β-actin in the presence (top 2 arrays) or absence of the primary antibody (bottom array).

**Figure 6:**
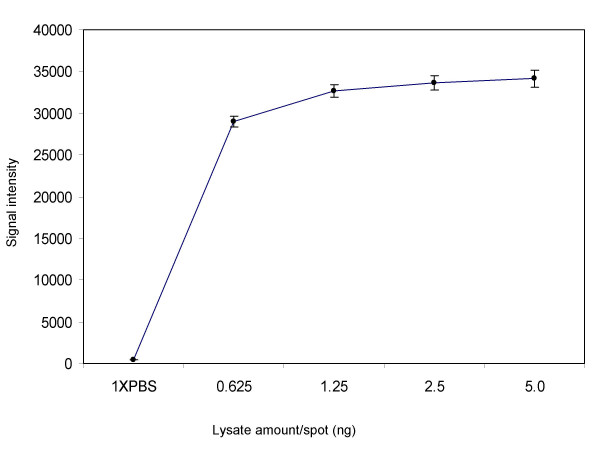
Correlation of signal intensity to lysate amount printed on LMA. Lysate amount printed on LMA (5.0 to 0.625 ng/spot in Figure 5) was plotted against signal intensity

**Figure 7:**
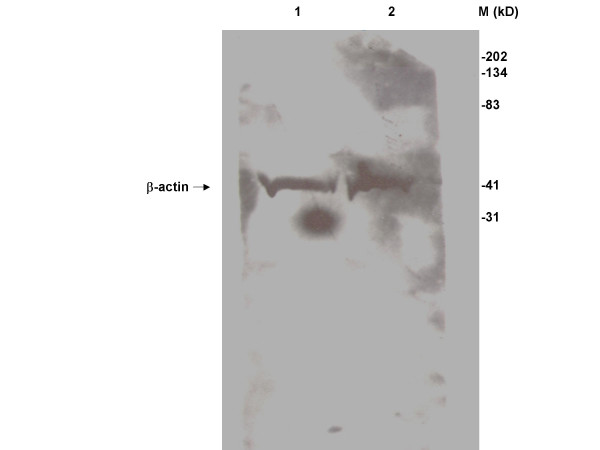
Specificity of β-actin antibody. Human plasma samples (lanes 1 and 2) were used to determine the specificity of the antibody for β-actin by Western blot analysis.

Serial dilutions of normal human plasma samples were printed onto membrane-coated glass slides. Lysate stocks used for printing were 0.625, 1.25, 2.5 and 5 μg/μl. The volume printed per spot by the robot is estimated to be 1 nl (Genomics Solutions, Inc. Irvine, CA). The printed amounts of lysates in each dilution series were 0.625, 1.25, 2.5 and 5 × 10^-9 ^g (nanograms; ng). Printing buffer (1X PBS) was used as a negative control. Each concentration of the plasma sample was printed 24 times per array and each experiment was performed in duplicate, resulting in a total of 48 data points per concentration. The arrays were incubated with an antibody against β-actin. Figure 5 (top) shows the two arrays, with serially diluted samples shown left to right in each row in each of the 4 sub-arrays in the 2 arrays. The printing buffer was spotted as negative control in the fifth column in each sub-array. A third array processed as a negative control where no primary antibody was added to the binding step, showed no discernable signals (Figure 5 bottom). The signals observed in this third array were close to background suggesting no binding by the secondary antibody in the absence of the primary antibody. Figure 6 shows a plot of correlation between signal intensity and lysate amount printed. Increase in the amount of printed lysate resulted in a correlative increase in the signal intensity generated. Table 2 shows a summary of the statistics of the data generated. For replicates of each sample spotted on the arrays, the mean was computed and SD and CV were determined. We observed a CV of 4.5–5.9% for the 48 data points per lysate amount printed. These data show that LMA technology can potentially be used to reproducibly detect proteins from extremely small amounts of lysate, less than a nanogram (625 picograms) for detection of β-actin from human plasma. Figure 7 shows a Western Blot where 2 different human plasma samples were probed for detection of β-actin by the same antibody that was used in the LMA experiments. The 40 kD unique band observed on the Western blot demonstrates the specificity of this antibody for β-actin.

**Table 2:** Summary of data statistics for LMA where printed lysate amount ranged from 0.625 to 5 ng.

	**1X PBS**	**0.625**	**1.25**	**2.5**	**5.0 (ng)**
MEAN	396.83	28992.14	32682.63	33619.77	34140.00
SD	27.67	1373.84	1486.66	1683.11	2013.82
CV (%)	6.97	4.74	4.55	5.01	5.90

Other proteins tested in similar experiments involved detection of tubulin from human plasma and insulin and leptin from diabetes serum samples (data not shown). In all cases, a linear correlation between signal intensity and lysate amount was observed and the data showed high reproducibility similar to the dataset presented here.

### Sensitivity of LMA

**Figure 8:**
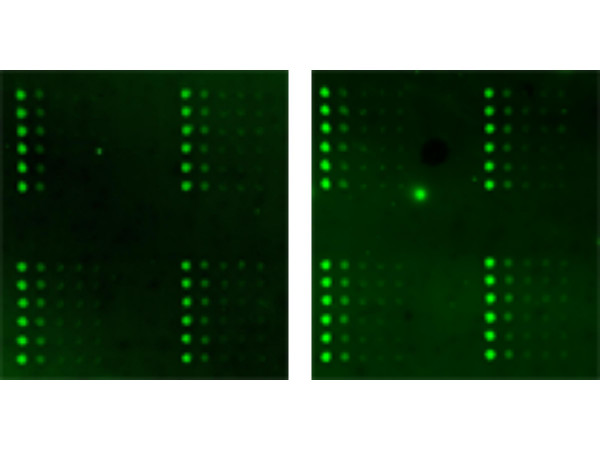
Sensitivity of LMA. Two arrays were printed using serial dilutions (6.66 to 0.006 pg) of human plasma sample and probed for β-actin.

**Figure 9:**
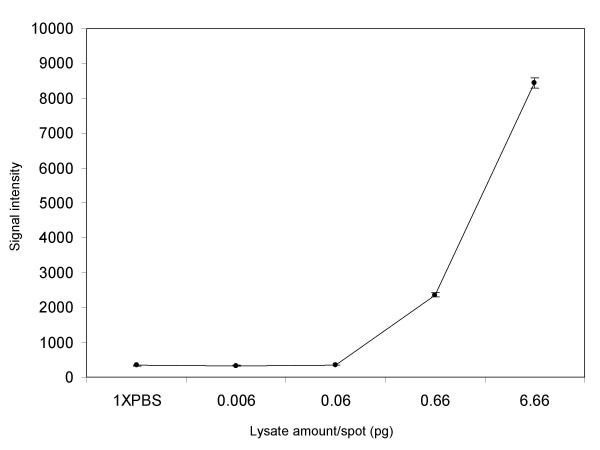
Correlation of signal intensity to lysate amount printed on LMA. Lysate amount printed on LMA (6.66 to 0.006 pg/spot in Figure 8) was plotted against signal intensity

LMA with human plasma lysate amounts ranging from 6 × 10^-12^g (6 picograms; 6 pg) to 6 × 10^-15^g (6 femtograms; 6 fg) were printed and probed for β-actin. Two arrays containing 24 replicates of each lysate amount were used for the experiment resulting in a total of 48 data points per concentration. Figure 8 shows the scanned images of the 2 arrays each with 4 sub-arrays. Serially diluted samples are shown left to right in each row in each of the 4 sub-arrays. The printing buffer was spotted as negative control in the fifth column in each sub-array. Figure 9 shows a plot of correlation between signal intensity and lysate amount. Summary of the data statistics is shown in Table 3. The data show that the mean signal intensity for lysate amounts lower than 0.6 pg was close to those generated by the printing buffer (1 × PBS in Figure 9), indicating these lysate amounts were not optimal for detection of β-actin from these samples using LMA. At lysate amounts, 0.6 and 6.66 pg the mean signal intensity was several fold higher (7-fold higher at 0.6 pg and 25-fold higher at 6.66 pg) than background and the data were reproducible with a CV on the order of 3.3–4.8% (Figure 9 and Table 3). These data show that β-actin can be reproducibility detected from less than a picogram (600 femtograms) of printed human plasma sample.

**Table 3:** Summary of data statistics for LMA where printed lysate amount ranged from 0.006 to 6.66 pg.

	**1XPBS**	**0.006**	**0.06**	**0.66**	**6.66 (pg)**
MEAN	334.50	333.67	344.78	2358.10	8443.57
SD	33.44	19.87	19.34	115.03	279.16
CV (%)	10.00	5.95	5.61	4.88	3.31

### Application of LMA: Verification of atherosclerotic protein biomarkers

In the series of experiments described here, the LMA technology was used to detect apolipoprotein [a] (apo [a]) and apolipoprotein B100 (Apo B) in samples from atherosclerotic patients. Apo [a] is contained exclusively in Lp [a] (lipoprotein [a]; 30–31), whereas Apo B is present in Lp [a], LDL (low density lipoprotein) and VLDL (very low-density lipoprotein; 32). Lp [a] is a plasma lipoprotein whose plasma concentration has been highly correlated with cardiovascular disease (e.g. coronary stenosis determined by quantitative angiography; 30–31). Furthermore, the plasma Lp [a] concentrations are significantly associated with tissue Lp [a] levels in resected bypass vein grafts and aortic aneurisms [30,31]. These observations suggest that Lp [a] may play a significant role in atherosclerosis occurring in other vascular beds such as the carotid arteries. Hence it was quite reasonable to measure Lp [a] in carotid plaques obtained by endarterectomy.

Since the Lp [a] lipoprotein contains one mole of apo [a] disulfide linked to one molecule of ApoB [33], it is possible to calculate the amount of Lp [a] from the amount of measured apo [a]. The ApoB, not linked to apo [a] in Lp [a], is assumed to be present in LDL. LDL is small enough to penetrate the intimal lining and accumulate in the subintimal space. Since VLDL is too large to accumulate in this space and become integrated into an atherosclerotic lesion, little if any of the ApoB present in these lesions can be attributed to VLDL. Thus, apo [a] and ApoB quantified together make it possible to estimate the amount of Lp [a] and LDL present in the atherosclerotic plaque.

In the carotid arteries, atherosclerotic plaque occurs predominantly at the bifurcation (B), but also at the internal (I) and common (C) segments. The external (E) segments are usually negative for plaque. Previously, ELISA has been shown to be an effective method for profiling the above proteins in atherosclerotic tissue samples [34].

LMA technology was used for verification of the presence of and quantification of these protein markers from the different segments (with and without plaque) of the carotid artery samples from 15 different patients (samples-1014, 1023, 1042, 1098, 1103, 1117, 862, 865, 893, 989, 900, 904, 912, 935 and 979). Lysates were prepared from 3–4 different segments of an endarterectomy specimen resected from the carotid artery for each patient (total of 52 samples). Serial dilutions of purified Lp(a) (4 picograms to 500 picograms) and LDL (20 picograms to 3 nanograms) were printed to prepare standard curves (supplementary data available) for estimating the amount of these proteins in the different samples. Each sample was printed in a dilution series of 5 steps and the printed lysate amount range was about 120 picograms to 180 nanograms. Two arrays each were probed with antibodies for β-actin, apo [a] and Apo B. Each dilution of each sample was printed in duplicate on each array resulting in 4 data-points per dilution per sample. Two arrays processed in the absence of a primary antibody did not generate discernable signals. The amount of signal generated in the arrays probed with apo [a] and Apo B antibodies were used to estimate the amount of the two proteins markers in the different sections for each patient with the help of the standard plots. The specificity of the antibodies for detection of apo [a] and Apo B has previously been demonstrated and published [34,35].

**Figure 10:**
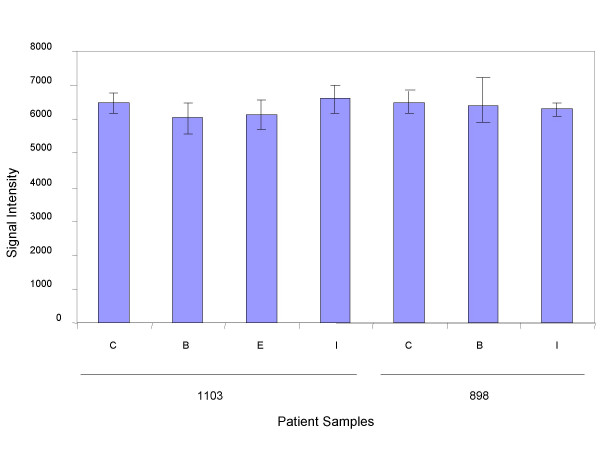
Detection of β-actin protein levels in atherosclerotic samples by LMA. Lysates prepared from sections C, B, E and I were analyzed for expression of β-actin by LMA. The average signal intensity for the different sections for 2 patients (samples-1103 and 898) is shown.

Cytoskeletal proteins such as β-actin may be differentially regulated during atherogenesis we determined its stability in these cases to establish β-actin as a viable control. In order to detect any variation in β-actin levels, the levels of β-actin in the different atherosclerotic samples were determined. The data for 2 patients (samples- 1103 and 898) is shown in figure 10. The average β-actin levels of the available samples from the different sections (C, B, I, E) are shown for each patient. No samples were available for segment E for patients 898. The data show no significant variation in the β-actin levels thus establishing this protein as a stable control in these samples valid for use as a means of normalizing the data. Similar data were generated for other patient samples.

**Figure 11:**
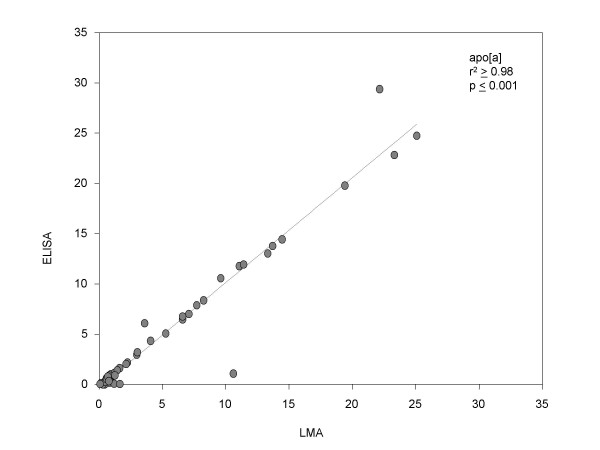
Comparison of apo [a] protein estimated by LMA and ELISA. Statistical correlation between the amount of apo [a] estimated (μg/ml) by LMA and ELISA.

**Figure 12:**
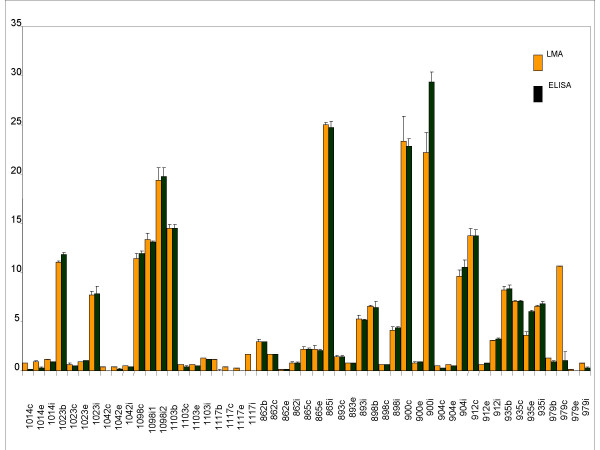
Comparison of apo [a] protein estimated by LMA and ELISA. Comparative histogram plot (x-axis: patient sample; y-axis: protein amount in μg/ml) for the estimated protein amounts by LMA and ELISA for all samples are shown.

In order to validate the data generated, aliquots of the same lysates (100 nanograms to 1 microgram) were used for verification of the presence and quantification of these proteins by ELISA, using the same set of antibodies. Standard plots were generated for ELISA (supplementary data available) using purified Lp(a) (1 nanogram to 10 nanograms) and LDL (10 nanograms to 140 nanograms) and used to estimate the amounts of these proteins present in the patient samples. Figures 11 (apo [a]) and 13 (Apo B) shows statistical correlation of the estimated protein amounts obtained using LMA and ELISA for the two protein markers. The correlations of the estimated protein amounts were highly significant with the r^2 ^≥ 0.98 (p ≤ 0.001) for apo [a] and r^2 ^≥ 0.94 (p ≤ 0.001) for apo B. Figures 12 (apo [a]) and 14 (Apo B) show comparative histogram plots for the estimated protein amounts in each section of each patient sample included in this study. The protein profiles for both proteins across the different sections varied between patients but were similar for LMA and ELISA for all the samples.

## Discussion

**Figure 13:**
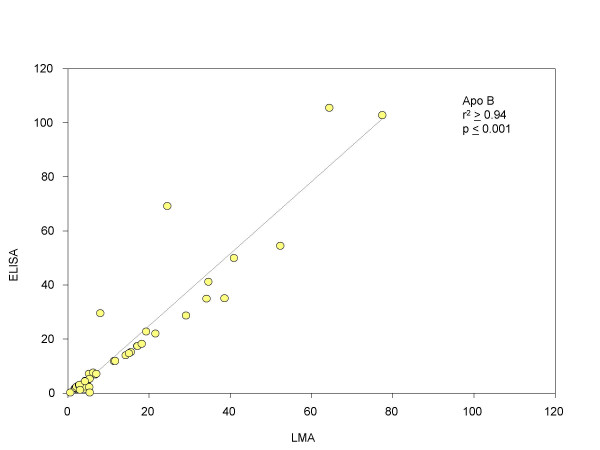
Comparison of Apo B protein estimated by LMA and ELISA. Statistical correlation between the amount of Apo B estimated (μg/ml) by LMA and ELISA.

Currently used methods for protein marker detection are based on classical immunological methods like ELISA that are not well-suited for high-throughput protein marker detection of human clinical tissue samples, where sample amounts are limiting, such as laser-capture microdissected sample or fine needle aspirate samples, due to the relatively large amount of sample required for the test. Microarrays offer a means of verification of the presence of protein markers in a high-throughput manner, typically in the order of thousands of samples in a single experiment, using much lower amount of sample than required for ELISA. We have systematically evaluated and optimized LMA for reproducibility in printing, linearity of the binding, labeling and detection steps and high sensitivity. The data presented here establish LMA as a potentially powerful technology for verification of the presence and quantification of known protein markers. This is the first comparative study of data generated by LMA and ELISA, showing a high correlation between the two data sets. The LMA reduces the amount of lysate sample required by as much as 1000-fold (nanogram versus picogram amounts) compared to ELISA. Our data showed a correlation coefficient, r^2 ^≥ 0.98 (p ≤ 0.001) for apo [a] (figure 11) and r^2 ^≥ 0.94 (p ≤ 0.001) for apo B (figure 13), between LMA and ELISA data.

**Figure 14:**
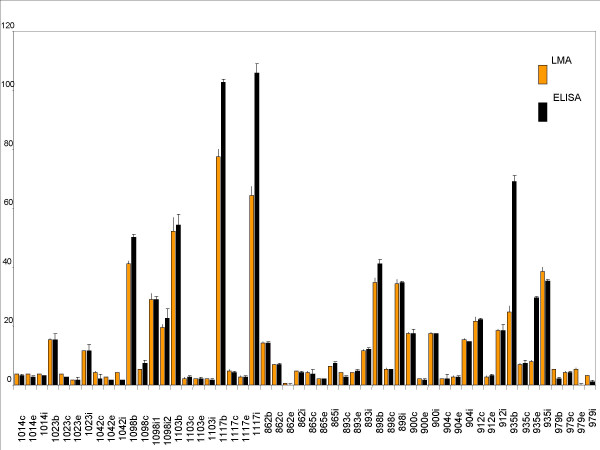
Comparison of Apo B protein estimated by LMA and ELISA. Comparative histogram plot (x-axis: patient sample; y-axis: protein amount in μg/ml) for the estimated protein amounts by LMA and ELISA for all samples are shown.

In very few cases the estimated protein amounts by LMA and ELISA were significantly different. We believe this may be due to the calcified nature of the carotid endarterectomy samples, which may alter the viscosity of the sample and thus may interfere with microarray printing. The presence of calcium in the samples, combined with the solid-pin printing technology (that is very sensitive to the level of viscosity of the sample) of microarrayer used for this study, might make it difficult for the calcified samples to be dispensed in a precise manner.

LMA offers an easy means of verification of the presence of protein markers in a high-throughput manner. The format of LMA, where the lysate is immobilized on the surface and probed with an antibody, enables high-throughput, and also reduces the amount of sample required since the lysate is not the limiting factor in the binding experiments. In LMA, the amount of antibody in solution exceeds the amount of lysate on the surface, and hence, the signals from binding are a true reflection of the amount of protein marker in the lysate. This is crucial to the comparison of protein markers across samples.

LMA with serially diluted purified proteins are used to prepare standard curves that can then enable quantification of the proteins of interest in the samples. In LMA, lysate samples are arrayed in a serial dilution thus yielding at least one or more data points in the dynamic range of the standard curve for the specific protein-antibody pair tested.

The sensitivity of detecting the presence of protein biomarker on LMA is greatly dependent on the signal detection and amplification method employed. In most of the studies performed using LMA so far an enzyme-mediated deposition of biotin-tyramide conjugates at the site of antibody binding has been used [36]. Although a sensitivity in the femtogram range has been achieved using this technology [21], it suffers from saturation at spots in LMA containing high amounts of the protein of interest, due to the inability to control the enzymatic reaction [28]. We have demonstrated detection of β-actin protein from human plasma samples from 600 femtograms (10^-15 ^gram) of protein lysate using a non-enzymatic signal detection method. Since β-actin is an abundant protein, we expect the LMA sensitivity for detection of less abundant proteins to be not as high.

The value and application of LMA is best understood in studies involving large clinical trials. In such clinical trials, the presence and the level of protein biomarkers (up to 10) in a group of patients (100–1000 or more) may need to be determined. The number of proteins is too small for an antibody array, and the throughput of the number of samples processed using antibody array is one patient sample at a time. If antibody arrays are used for this study, 100–1000 or more arrays will be required (one array per patient sample) whereas *with LMA this study can be completed with 10 arrays with all patient samples arrayed on every single array and probing with one antibody per array*. LMA would also be a useful technology to use when a large number of archived samples (thousands) need to be interrogated for a predefined set of protein markers.

Using ELISA, the amount of starting material (lysates) required is at least 10 times higher than LMA and the throughput of number of samples processed using ELISA is only 96 per plate (8–200 samples per experiment using SELDI-TOF MS). Using LMA, up to 4000 patient samples can be assayed for the detection of a single protein, simultaneously in a single experiment, using simple means of arraying, hybridization, and imaging, while maintaining quality controls at each step. Since the amount of sample required per spot is only a few picograms, many proteins can be estimated (albeit one at a time) using LMA. Thus, LMA offer a faster, easier and cheaper (single slide in LMA versus many slides in antibody arrays) method to study for large clinical trials.

One essential requirement for the application of LMA as a high-throughput protein biomarker tool is to be able to process and generate protein lysates from patient samples in an equally high-throughput manner. This can be achieved by minimal customization of any of the several liquid-handling robotic set-ups commercially available today. The protocols adopted for lysate generation will be dependent on the source of the samples, such as, serum, biopsies etc. With current capacity of robotics hundreds of samples could be lysed directly in microtitre plates and prepared for printing LMA, which can then serve as input plates for a printing robot, thus requiring minimal sample handling.

## Conclusion

We have demonstrated the use and applicability of lysate microarray technology for detection and quantification of pre-determined protein markers. The high sensitivity (reduces amount of input sample required) and miniaturized format (increases the number of samples assayed on a single slide) of LMA technology, makes it suitable for studies such as large clinical trials where thousands of samples (available in limited quantities) may need to be interrogated for the expression level of a defined set of protein markers. LMA can be used for estimating the amount of specific proteins present in these samples by generating standard plots prepared using corresponding purified proteins arrayed on the LMA. The comparative data analysis of LMA and ELISA data show that the data were highly correlative (in over 90% of the samples) indicating that LMA can potentially be used as a diagnostic tool for verification of the presence and quantification of specific protein markers in clinical samples where the sample amounts are limiting.

## Methods

### Purified proteins, antibodies, cell culture and patient samples

Purified p53 protein (sc-4246, human) and p53 (C-19, goat polyclonal), were purchased from Santa Cruz Technologies (Santa Cruz, CA). The antibody for β-actin (AC-15, monoclonal) was purchased from Sigma-Aldrich (St. Louis, MO). Polyclonal antibodies for apo [a] and Apo B were raised in goat and purified as described previously [34,35]. Biotinylated secondary antibodies were purchased from Vector Laboratories (Burlingame, CA).

F9 cells were cultured in Dulbecco's Modified Essential medium (Invitrogen Life Technologies, Carlsbad, CA) supplemented with 10% Fetal Bovine Serum. All cells were grown to 80% confluency in 10 cm plates at 37°C. Cells were washed twice in 1XPBS and cell pellets were stored at -70°C until further use. Lysates from bacteria over expressing p53 protein were a gift from Dr. Guillermina Lozano [37]. Normal human plasma and surgical tissue samples were obtained under a protocol approved by the Human Research Committee of Baylor College of Medicine, Houston, TX.

### Tissue processing

Tissue samples were obtained at endarterectomy of atherosclerotic carotid arteries from patients enrolled in a cardiovascular study at the Methodist Hospital (Houston, TX) and stored immediately after surgery in PBS containing 50% glycerol at -20°C until imaged or analyzed. Slits in the carotid artery tissue created at surgery were closed by annealing opposing sides with superglue so as to recover the original spatial relationships within the tissue. The tissue was x-rayed using a FAXITRON analyzer to identify areas of calcification and guide subsequent dissection. After magnetic resonance imaging (MRI), the tissue was marked with a thin line of India ink along the long axis. Transverse segments 3 mm long (corresponding to the MRI slice thickness) were cut with a scalpel, starting at the bifurcation and extending into the common, internal and external areas of the arteries. Segments were inked on the proximal end to preserve the correct end-to-end relationship. Segments (100 mg) were then transferred to 15 ml plastic tubes containing 3 ml of extraction buffer (50 mM Hepes, pH7.4, 150 mM NaCl, 1 mM EGTA, 10 mM sodium pyrophosphate, 100 mM NaF, 1.5 mM MgCl2, 10% Glycerol, 1% Triton X-100, pH 7.4) then homogenized at 10°C for 10–20 sec (Polytron, Brinkmann). The homogenate was centrifuged at 3500 rpm for 30 min and the supernatant decanted for storage at -80°C until analyzed.

### Cell Lysate Preparation

The pellets from F9 cells and patient samples were suspended in extraction buffer (as above) containing 0.5X protease inhibitor cocktail (Roche Diagnostics, Mannheim, Germany). The cell lysate was incubated on ice for 30 min followed by centrifugation at 4°C, 14,000 rpm for 10 min. The supernatant was transferred to a fresh tube. The protein concentration was measured using the Bradford assay (Bio-Rad, Hercules, CA) as per manufacturer's instructions. The proteins were solubilized by addition of one-third volume 4X SDS buffer (0.25 M Tris.HCl, pH 6.8, 40% glycerol, 8% SDS and 10% β-mercaptoethanol) and denatured at 94°C for 5 min. Aliquots of extracts containing denatured proteins were stored at -70°C. Plasma samples and purified proteins were directly denatured in 4X SDS buffer as above.

### Array manufacture and Processing

The lysates were serially diluted in 1 X phosphate-buffered saline (PBS, Invitrogen, Carlsbad, CA), to desired concentrations and transferred to a 384 well plate (20 μl/well; Fisher Scientific, Pittsburg, PA). The lysates were printed onto membrane-coated glass slides (FAST slides, Schleicher & Schuell Bioscience, Keene, NH) using the Flexys robot (Genomic solutions, Ann Arbor, MI). Flexys is a solid pin robot with an estimated printed amount of 1 nl per spot (Genomics Solutions, Inc. Irvine, CA). All samples were printed in a dilution series of 4 or more steps. Two or more replicates of each sample concentration were printed per array and each experiment set included 2 or more arrays (2 or more slides). The arrays were stored at 4°C until further use.

Non-specific binding sites on the arrays were blocked by addition of 10 ml of 1 X Wash/Block buffer (Fast Pak2 protein array kit, Schleicher & Schuell Bioscience, Keene, NH) and incubation with gentle rocking, for 2 h at room temperature. The excess buffer was drained off without allowing the arrays to dry. This was followed by binding with 50 μl of primary antibody at a concentration of 10 ng/μl in 1XPBS containing 0.5% Tween-20 (Sigma-Aldrich, St. Louis, MO) and 5% bovine serum albumin (BSA, Sigma-Aldrich, St. Louis, MO). The array was covered with a cover slip and incubated in a humid chamber overnight (15–16 hr) at 4°C. Slides were rinsed in 1 X Wash/Block buffer for 5 min with gentle rocking at room temperature to remove the cover slip. Each slide was carefully removed from the Wash/Block buffer letting the excess buffer drain off without allowing the arrays to dry. Next, 50 μl buffer (50 mM Tris pH 7.5, 1.5 M NaCl, 0.1% Tween-20) containing biotinylated secondary antibody (Vector Laboratories, Burlingame, CA) at a concentration of 5 ng/μl was added to the arrays and a cover slip was placed on the arrays. The arrays were incubated in a humid chamber at room temperature for 1 h.

Slides were rinsed in 1 X Wash/Block buffer for 5 min with gentle rocking at room temperature to remove the coverslip. Slides were removed from the wash/Block buffer letting the excess buffer drain off without allowing the arrays to dry. Cyanine 3/Cyanine 5 (Cy3/Cy5)-labeled streptavidin dyes (Amersham Biosciences, Piscataway, NJ) were reconstituted in water to a stock of 1 mg/ml. Next, 50 μl of the Cy3/Cy5-labeled streptavidin (1:3 (v/v) in 1XPBS) was added to the array and covered with a cover slip. The arrays were incubated in a dark humid chamber at room temperature for 1 h. Slides were rinsed in 1 X Wash/Block buffer for 5 min with gentle rocking at room temperature to remove the cover slip. The slides were removed from the wash tray, placed in a 50 ml tube and dried by centrifugation at 1.5 rcf for 90 sec in a clinical centrifuge.

Monofunctional NHS-esters forms (for labeling compounds containing free amino groups) of Cy3 and Cy5 monoreactive dyes (Amersham Biosciences, Piscataway, NJ) were used to label protein lysates. 100 μl of 0.1 M sodium carbonate buffer (pH 8.0) was added to each of the Cy3 and Cy5 monoreactive dyes (to yield a stock of 10 mg/ml) and 25 μl of the dye (Cy3 or Cy5) was added to different amounts of protein/lysate to be labeled. The final volume of the solution was adjusted to 35 μl using 0.1 M Sodium carbonate buffer (pH 8.0). The protein-dye mixture was incubated at 4°C for 1 hour and agitated gently every 10 min. This was followed by the addition of 3.5 μl of 1 M Tris.HCl (pH 8.0) and 1XPBS to give a final volume of 500 μl. The protein solution was concentrated by passing it through a micro concentrator spin column (Amicon-Microcon YM-10 column, Millipore, Billerica, MA) and centrifugation at 14000 rcf for 25 min, reducing the volume to 30 μl. This step also facilitated the removal of unincorporated dye molecules from the labeled protein solution.

### ELISA

ELISA was performed as described by us previously [29]. Briefly, 100 μl of serially diluted purified Lp(a) or LDL (starting with a stock of 1 mg protein/ml in PBS, pH7.2) were added to 96-well microtitre plates (Dynatech Labs, Alexandria, VA) and incubated for 2 h at room temperature (RT). Two wells were left uncoated to serve as controls. The plates were washed successively with PBS-BSA (PBS containing 0.5% BSA) and PBS. Plates were incubated with PBS-BSA for 2 h at RT, then overnight at 4°C. Appropriate dilutions (prepared in PBS-BSA) of homgenate supernanants prepared from atherosclerotic tissue samples were mixed with the antibody against apo [a] or Apo B (1: 5000 dilution) and added to the plates. Antibody diluted in PBS-BSA was added to the uncoated wells. Plates were incubated for 2 h at RT, then overnight at 4°C. Plates were washed as before and incubated with a peroxidase-conjugated secondary antibody that is immunoreactive against the IgG species (anti-IgG-HRP; 1: 2500 dilution; Cappel labs, Cochranville, PA), followed by incubation at RT for 2 h and washing. Substrate solution was prepared by dissolving 10 mg of o-phenylenediamine in 25 ml of phosphate-citrate buffer (24.3 mM citrate, 51.2 mM phosphate, pH 5.0) containing 10 μl of 30% H_2_O_2_. Next 160 μl of substrate solution was added to each well, the reaction carried out for 30–60 min at RT and then quenched by addition of 40 μl of 2.5 N sulfuric acid. Absorbance at 492 nm was measured using a plate reader (Titertek Multiskan Plus MKII microtitre plate reader, Flow Laboratories). The absorbance values of samples were equated to Lp [a] or LDL protein using standard curves generated with purified lipoproteins, whose protein content had been previously measured by Bradford assay (Bio-Rad, Hercules, CA).

### Western Blot Analysis

Thirty micrograms of denatured lysates were subjected to 10% sodium dodecyl sulfate-polyacrylamide gel electrophoresis. After electrophoresis, Western blotting was performed using Hybond nitrocellulose membranes (Amersham, Piscataway, NJ) as per manufacturer's protocols. After protein transfer, the membranes were washed and blocked in 1XPBS buffer containing 0.3% Tween 20 and 5% milk for 15 min at RT and incubated with respective primary antibody (at 1:1000 dilution in 1XPBS containing 0.3% Tween 20 and 5% BSA) overnight at 4°C on a shaker. The membranes were washed 3 times with PBST (1XPBS containing 0.3% Tween 20) for 10 min at RT each time and subsequently incubated with the respective secondary antibody (1:1000–3000 dilution of HRP-conjugated anti-rabbit IgG, anti-mouse IgG or anti-rabbit IgG in 1XPBS containing 0.3% Tween 20 and 5% BSA; Amersham Biosciences, Piscataway, NJ) for 1 h at RT with shaking. The blots were washed with PBST as before and the protein bands were detected using ECL Plus Western Blotting Detection Reagents (Amersham, Piscataway, NJ).

### Imaging and Data Analysis

All microarrays were imaged at 10μ resolution using a dual laser-scanner (GenePix 4000; Axon Instruments, Union City, CA) to capture fluorescence intensity signals generated for Cy3 at 532 nm or Cy5 at 635 nm. Image acquisition and quantification were performed using GenePix Pro 4.1 (Axon Instruments, Union City, CA). The signal intensity at each spot was determined by subtracting the median intensity of the background pixels from the mean intensity of the foreground pixels. These background-corrected intensity values were truncated by replacing negative or zero values by the threshold value of 1.

For some analyses, the signal intensities were transformed by computing the logarithm (base two). For replicates of each sample spotted on each array, mean, standard deviation (SD) and coefficient of variance (CV) were calculated. To analyze the data for determining the performance of different binding reactions, we fitted a Generalized Linear Mixed Model with common slope for log concentration, fixed effect for the specific binding step with Cy3 as the reference group and random effect for array. The response was log intensity. Since the intensity was base two logarithmic transformed, two to the difference power of the intercepts between the different reactions was the ratio of the intensities, which represented the relative amount of the signal for the specific reaction. We used Cy3-streptavidin as the reference and calculated the ratios of other reaction signals based on it. This resulted in the relative signal for the specific signal as compared to Cy3.

For the comparing the data generated using ELISA and LMA, same set of protein lysates were used for LMA and ELISA experiments. Standard plots were generated using purified proteins for both technologies. The standard plots were used to estimate the amount of the specific proteins in each sample. SigmaPlot (Statistical Solutions, Saugus, MA) was used for determining the correlation between the protein amounts estimated in each sample by LMA and ELISA experiments.

## List of Abbreviations

LMA (lysate microarrays), ELISA (enzyme-linked immunosorbent assay), apo [a] (apolipoprotein [a]), LDL (low density lipoprotein), Apo B (apolipoprotein B)

## Competing interests

The author(s) declare that they have no competing interests.

## Authors' contributions

AR performed the microarray experiments, imaged and quantified the data. EL participated in the data analysis. IC performed the ELISA on the atherosclerotic samples. RM participated in the design and analysis of the atherosclerotic sample data. JM provided the atherosclerosis samples and coordinated the ELISA experiments. KC participated in the data analysis. ZJ performed the Western blot analysis. MK conceived of the study, participated in the design and drafted the manuscript. All authors read and approved the final manuscript.
